# Inborn Errors of Fructose Metabolism. What Can We Learn from Them?

**DOI:** 10.3390/nu9040356

**Published:** 2017-04-03

**Authors:** Christel Tran

**Affiliations:** Center for Molecular Diseases, Division of Genetic Medicine, Lausanne University Hospital (CHUV), Beaumont-02/248, Lausanne CH-1011, Switzerland; christel.tran@chuv.ch; Tel.: +41-79-556-5325; Fax: +41-314-3546

**Keywords:** fructose, inborn errors of metabolism, toxicity

## Abstract

Fructose is one of the main sweetening agents in the human diet and its ingestion is increasing globally. Dietary sugar has particular effects on those whose capacity to metabolize fructose is limited. If intolerance to carbohydrates is a frequent finding in children, inborn errors of carbohydrate metabolism are rare conditions. Three inborn errors are known in the pathway of fructose metabolism; (1) essential or benign fructosuria due to fructokinase deficiency; (2) hereditary fructose intolerance; and (3) fructose-1,6-bisphosphatase deficiency. In this review the focus is set on the description of the clinical symptoms and biochemical anomalies in the three inborn errors of metabolism. The potential toxic effects of fructose in healthy humans also are discussed. Studies conducted in patients with inborn errors of fructose metabolism helped to understand fructose metabolism and its potential toxicity in healthy human. Influence of fructose on the glycolytic pathway and on purine catabolism is the cause of hypoglycemia, lactic acidosis and hyperuricemia. The discovery that fructose-mediated generation of uric acid may have a causal role in diabetes and obesity provided new understandings into pathogenesis for these frequent diseases.

## 1. Introduction

People in developed countries may ingest up to 50 to 100 g fructose equivalents daily in their diet and the use of this sugar in foods and drinks is increasing globally [1,2]. Fructose is almost exclusively derived from the diet. It is found in its free form in honey, fruits, and many vegetables, and is associated with glucose in the disaccharide sucrose in numerous foods and beverages. Sorbitol, widely distributed in fruits and vegetables, is converted to fructose in the liver by sorbitol dehydrogenase. Recently it has been demonstrated that fructose is also produced endogenously from glucose by the human brain via the polyol pathway [3]. Exogenous fructose is absorbed through glucose transport proteins (GLUT) 5 and 2 across the intestinal epithelium and is metabolized (mainly in the liver) by the enzymes fructokinase, aldolase B, and triokinase. The majority of the fructose is converted into glucose, which can be either stored as glycogen or released as plasma glucose. Part of the fructose is oxidized or converted into lactate and fatty acids through the process of de novo lipogenesis [4]. Dietary sugar has particular effects on those whose capacity to metabolize fructose is limited. Three inborn errors are known in the pathway of fructose metabolism; (1) essential or benign fructosuria due to fructokinase deficiency; (2) hereditary fructose intolerance (HFI); and (3) fructose-1,6-bisphosphatase (FBPase) deficiency. The description of the clinical symptoms and biochemical anomalies in the three inborn errors of metabolism (IEM) is preceded by an outline of the metabolism of fructose [5]. The potential toxic effects of fructose in healthy humans also are discussed: it is essential to understand these toxic effects in order to comprehend the pathophysiology of HFI and of FBPase deficiency.

## 2. An Overview of Fructose Metabolism

### 2.1. Enzyme of Fructose Metabolism

**Figure 1:**
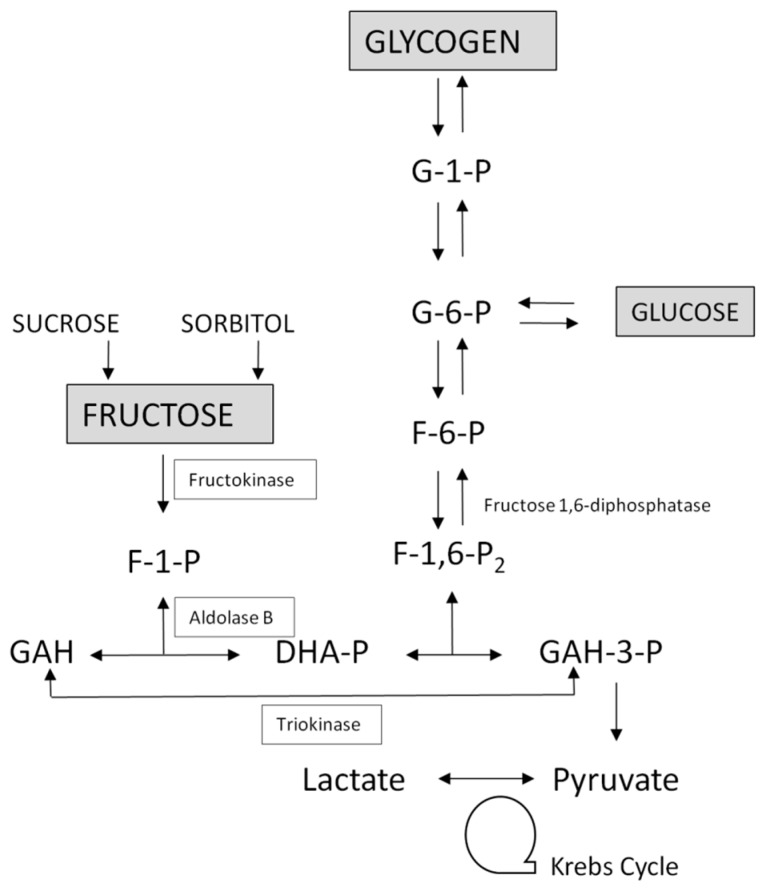
Fructose metabolism. Fructose enters the cell via the fructose transporter GLUT5 and GLUT2. Liver enzyme fructokinase phosphorylates fructose to fructose-1-phosphate. This is cleaved by fructose-1-phosphate aldolase (aldolase B) to form dihydroxyacetone phosphate and glyceraldehyde. Glyceraldehyde is then phosphorylated by triokinase to glyceraldehyde-3-phosphate. Thus, the intermediary metabolites of fructose enter glycolysis and the Krebs cycle as triose phosphates. Adapted from [10] and [11]. F: fructose; G: glucose; P: phosphate; DHA: dihydroxyacetone; GAH: glyceraldehyde.

Fructose utilization in humans and animals occurs mainly in the liver, kidney, and small intestine [6]. Unlike glucose, fructose can enter muscle cells and adipocytes in the absence of insulin by using facilitative GLUT. However, glucose can enter muscle and adipose tissue in the absence of insulin albeit in very small quantities. After apical transport mediated by GLUT5, fructose is transported across the basolateral membrane by GLUT2 [7]. The predominance of liver, kidney, and small intestine in fructose metabolism is based on the presence of the three enzymes—fructokinase, aldolase type B, and triokinase—which convert fructose into intermediates of the glycolytic–gluconeogenic pathway (Figure 1) [8]. Both fructose and glucose can be degraded into triose-phosphate and lactate and, thus, yield glycolytic intermediates. Their two initial metabolic steps, however, differ: fructose at physiological concentration is not readily phosphorylated by hexokinase, the enzyme catalyzing the synthesis of glucose-6-phosphate from glucose in all cells of the organism; instead, it is first phosphorylated to fructose-1-phosphate (F-1-P) by a specific enzyme, fructokinase, then converted into triose-phosphate by a second enzyme, aldolase B [9]. These apparently minor metabolic variations, however, have profound metabolic consequences, as discussed further.

### 2.2. Fructose Toxicity

After the discovery of HFI, fructose toxicity was thought to be limited to individuals with the aldolase B defect. In the late 1960s, deleterious effects of high dose intravenous (IV) fructose were also recognized in healthy persons. Hyperuricemia [12] and lactic acidosis [13] were the prominent findings and are due to the influence of fructose on purine catabolism and glycolytic pathway, respectively. These observations have led to the recommendation of great caution when using fructose in parenteral nutrition [14,15]. IV fructose bypasses the regulatory steps that control glucose catabolism in the following ways: (1) entry of fructose into cells is insulin-independent; (2) IV feeding with large quantities of fructose depletes cellular inorganic phosphate and lowers the concentration of ATP; and (3) in liver, fructose evades the rate-limiting control mechanism by entering glycolysis as dihydroxyacetone phosphate or glyceraldhehyde 3-phosphate [10]. Resulting accumulation of F-1-P provokes important changes in the concentration of several other metabolites (e.g., glucose, lactate, and uric acid) which explains the toxic effects of fructose. In hypoxic conditions, such as shock or severe trauma, IV fructose may cause a massive unregulated flux of metabolites through glycolysis and fatal lactic acidosis [16]. Fructose toxicity is not only limited to the IV route as, orally, fructose consumption can also be deleterious when consumed in large quantities in the daily diet. In recent years, increased consumption of fructose, particularly from sweetened beverages, has been associated with an increased prevalence of obesity, metabolic syndrome, type 2 diabetes, and gout [4,17]. Compared to other nutrients, such as glucose or fat, fructose is first processed in the splanchnic organs and then released as glucose, lactate or VLDL-TG into the systemic circulation. These products, in turn, promote intrahepatic fat deposition, hepatic insulin-resistance, hypertriglyceridemia, hyperuricemia, and hypertension. These fructose-induced effects were proposed as being markers of cardiometabolic diseases. Nevertheless some controversies exist on how, and to what extent, those changes alter cardiovascular risk [18].

## 3. Inborn Errors of Fructose Metabolism: A Model for Fructose Toxicity

Inborn errors of fructose metabolism are summarized in Table 1.

### 3.1. Essential Fructosuria-Hepatic Fructokinase Deficiency (OMIM 229800)

This rare and benign error of metabolism was first described in 1876. The disorder is caused by the inherited deficiency of fructokinase. Since the disorder is asymptomatic and harmless, many cases may remain undetected and the detected ones unpublished. Prevalence is, therefore, unknown. Ingested fructose is partly excreted unchanged in the urine and the rest is metabolised by an alternative pathway, namely, conversion to fructose-6-phosphate by hexokinase in adipose tissue and muscle [19]. Affected persons are usually discovered on routine urinalysis by the presence of reducing sugars with a negative reaction with glucose oxidase. The misdiagnosis of diabetes is avoided only when the non-glucose nature of the sugar is recognized. After an oral or IV load of fructose (1 g/kg body weight), blood fructose increases rapidly, far the level seen in controls and declines slowly. Between 10% and 20% of the administered dose is excreted in the urine as compared with 1%–2% in normal subjects [20]. The loss of fructose into the urine in this condition illustrates well the fact that fructose, having escaped hepatic metabolism, is poorly metabolized in extrahepatic tissues. Using ^31^P magnetic resonance spectroscopy (MRS) to measure changes in liver metabolite concentrations in adults with fructosuria, Boesiger et al. found that concentrations of F-1-P, ATP, and inorganic phosphate remained unchanged, confirming that fructokinase was indeed inactive [21]. Heterozygotes appear to excrete no more fructose after an oral load than normal subjects [22]. An oral load with 50 g of fructose produces a further increase of fructose-3-phosphate in the red cells. Erythrocytes were shown to metabolize fructose to fructose-3-phophate. Concentrations of fructose-3-phophate were 3 to 15 times higher in adult with essential fructosuria than in healthy controls [23]. Accumulation of fructose-3-phosphate in the lens of diabetic rats raised the hypothesis of a diabetogenic effect of fructose-3-phophate [24]. Nevertheless, neither cataracts nor diabetes have been reported in subjects with essential fructosuria and is likely of a benign nature.

### 3.2. Hereditary Fructose Intolerance and Accumulation of F-1-P (OMIM 229600)

HFI is caused by a deficiency of the second enzyme of the fructose pathway, aldolase B which splits F-1-P into dihydroxyacetone phosphate and glyceraldehyde in the liver, small intestine and proximal renal tubule (Figure 1). The triose products of aldolase B are key intermediates in glycolysis and gluconeogenesis (GNG), but many of the manifestations of HFI are attributable to the toxicity of non-degraded F-1-P. Because of the high activity of fructokinase, intake of fructose results in accumulation of F-1-P and the trapping of phosphate. This has two major effects: (1) Inhibition of glucose production by blockage of GNG and of glycogenolysis which induce a rapid drop in blood glucose and (2) overutilization and diminished regeneration of ATP. This depletion of ATP results in increased production of uric acid and a release of magnesium as well as impaired protein synthesis and ultrastructural lesions which are responsible for hepatic and renal dysfunction. The toxic effects of fructose in HFI can be fatal. Patients with HFI only develop symptoms when they are exposed to fructose either as the monosaccharide, or in sucrose or sorbitol. Vomiting, diarrhea, abdominal pain, hypoglycemia, hepatomegaly, jaundice, and renal failure can ensue. IV administration of fructose to healthy subjects also induces the metabolic derangements described above (including the drop in ATP and inorganic phosphate and the rise of urate and magnesium) to an equivalent extent, although they are more transient than in patients with HFI, as demonstrated by ^31^P-MRS [21]. The similarity between HFI and IV fructose in healthy persons is striking, indicating the importance of upstream control on fructose rate entry in the cells which is dependent on blood fructose concentration. This is even more underscored when information about essential fructosuria is added, indicating that unmetabolized fructose is innocuous, while fast partial metabolism in the liver seems to be the genesis of fructose toxicity. Diagnosis of HFI is suspected from a detailed nutritional history and the clinical picture. As soon as HFI is suspected, all sucrose, fructose, and sorbitol must be eliminated from the diet and medications. It should be remembered that sucrose and sorbitol, often used as excipients in pediatric formulations, are not always harmless and may trigger metabolic decompensation in patients not yet diagnosed with HFI [25]. Prognosis is excellent and recovery within a few days after fructose eviction. Diagnosis is confirmed by molecular analysis of the *ALDOB* gene. If no mutation can be found despite a strong clinical and nutritional history suggestive of HFI, demonstration of deficient (<10%) aldolase activity in liver sample will confirm the diagnosis. In the past IV fructose tolerance test has served as a functional method of diagnosing HFI [26]. However due to the risk of severe and eventually fatal adverse metabolic effects induced by fructose infusion and the lack of availability of D-fructose for IV use, this test is no longer carried out.

Few studies have examined the effect of fructose ingestion in heterozygotes subject for HFI. HFI prevalence in central Europe is estimated to be 1:26,100 [27]. Based on this, a carrier frequency is predicted between 1:55 and 1:120 [28]. Although heterozygous carriers have no reported metabolic defects, they may have enhanced uric acid responses to IV and oral fructose load (50 g) according to some reports [26,29]. Assuming that estimated oral fructose consumption might reach 50 g/day in the United States, heterozygous carriers may be predisposed to gout [4]. Speculation about fructose intake and gout was raised about 50 years ago [12], and in some patients with gout hyperuricemia, the frequency of gouty attack was reduced by a fructose restricted diet [30]. It seems possible that those who benefited were heterozygous for HFI. Recently a meta-analysis of prospective cohorts studies investigating total fructose consumption and its association with incident hyperuricemia and gout concluded with a significant overall association. However the strength of evidence for the association between fructose consumption and risk of gout was low, meaning that further prospective studies are needed to conclude on which extent fructose may mediate the risk of hyperuricemia and gout [31,32].

There is evidence from mouse model that high amount of fructose consumption can also lead to non-alcoholic fatty liver disease (NAFLD) in the liver and obesity [33]. The recent generation of a mouse model (*Aldo2*^−/−^) clearly phenocopies the human condition of HFI, which might provide a valuable resource for answering remaining questions about fructose metabolism and liver damage [34].

### 3.3. Fructose-1,6-Bisphosphatase Deficiency (OMIM 229700)

FBPase deficiency is an autosomal recessive disorder and impairs liver formation of glucose from all gluconeogenic precursors, including dietary fructose. Its frequency is much lower than HFI and estimated to be around 1–9/100,000 [35]. Less than 100 patients are described and very few affected adults have been reported. FBPase is a key enzyme of GNG. Its inactivity prevents the endogenous formation of glucose from the precursor lactate, glycerol, and gluconeogenic amino acids, such as alanine. Infants become symptomatic when they are dependent on GNG within the first weeks of life [36]. During fasting, maintenance of blood glucose depends on glycogenolysis, and the duration of normoglycemia thus depends on the amount of available liver glycogen. When hypoglycemia is reached, the blood glucose level is unresponsive to injected glucagon and the defect provokes an accumulation of gluconeogenic substrates (lactate, alanine, and glycerol) [37]. Investigation of fasting GNG in a patient with liver-and kidney specific FBPase deficiency, using an in vivo stable isotopes dilution method, showed that GNG contributed to up to 20% of whole body glucose endogenous production, raising the hypothesis of a specific FBPase isoform activity in muscles. This observation contributed to the knowledge of the potential role of extrahepatic and extrarenal tissue in glucose homeostasis during fasting [38]. Tolerance to fructose in FBPase deficiency is higher than in patients with HFI. ^31^P-MRS of the liver following IV fructose (200 mg/kg BW) administration documented a slower decrease in the F-1-P accumulation and a delayed recovery of the ensuing depletion of Pi and ATP in adult patients with FBPase deficiency relative to healthy controls [21]. Although in FBPase deficiency, fructose changes of metabolite is less pronounced than in HFI, fructose tolerance test are not without risk and severe neurological complications has been reported following the administration of fructose or glycerol [39,40]).

Childhood manifestations of FBPase deficiency include hypoglycemia and lactic acidosis. Episodes are triggered by catabolic triggers, such as fever, diarrhea, and prolonged fasting. Metabolic disturbances seem to diminish with increasing age and adult patients are more tolerant of catabolic stressors, as well as sugar intake (sorbitol, fructose, or glycerol ingestion), with the exception of pregnancy, which is a serious risk factor for metabolic decompensation, due to its increased glucose requirements. Diagnosis of FBPase deficiency is established by molecular analysis of the *FBP1* gene and eventually FBPase activity in liver samples if no mutation is found [41]. Prognosis is excellent as long as metabolic decompensation is prevented by avoidance of prolonged fasting, as well as fructose and sucrose restriction in the diet. Hypoglycemia should be treated properly with the administration of exogenous glucose or by glucose infusion. Pre-pregnancy education and self-monitoring of blood glucose to prevent hypoglycemia, as well as continuous glucose infusion during fasting delivery, reduce maternal and fetal complications [42,43].

Sometimes rare diseases serve as a model for developing therapeutic strategies for more common diseases; in the case of FBPase, being a gluconeogenic enzyme, the role of selective inhibitors of FBPase on glucose control has been raised as a potential drug therapy for type 2 diabetes [44]. In animal models of type 2 diabetes, inhibition of FBPase effectively lowers endogenous glucose formation without causing hypoglycemia [45]. Preliminary results in 42 patients with type 2 diabetes demonstrated a modest glucose-lowering effect [46]. Some phase 2 clinical studies are in progress [47]. Safety concerns about hypoglycemia and lactic acidosis may limit the clinical usefulness of FBPase inhibitors.

## 4. Conclusions

Studies conducted in patients with inborn errors of fructose metabolism helped to understand fructose metabolism and its potential toxicity. Influence of fructose on the glycolytic pathway and on purine catabolism is the cause of hypoglycemia, lactic acidosis, and hyperuricemia under certain conditions and in certain populations. Toxicity was first thought to be limited to individuals with inborn errors of fructose metabolism, however, a few decades thereafter, deleterious effects of fructose were also recognized in healthy persons when exposed to large quantities of IV or oral fructose. Following those studies, there has long been interest in the metabolic effect of dietary fructose. The discovery that fructose-mediated generation of uric acid may have a causal role in diabetes and obesity provided new understandings into pathogenesis for these frequent diseases. Nevertheless, the contribution of those effects on cardiovascular risk remains unclear and many mechanisms still need to be clarified before drawing definitive conclusions. Future IEM research can yield important insight into more common conditions. Additionally, rare diseases are often more extreme and have a more straightforward etiology than their common counterparts and, therefore, provide models of disease that are easier to study. Rare diseases are fundamental to understanding common diseases.

## Figures and Tables

**Table 1:** Summary of enzyme defect, main clinical symptoms, and treatment of inborn errors of fructose metabolism.

Name of the Disease	Enzyme Defect	OMIM Number	Main Clinical Symptoms	Gene Mutations/Inheritance	Treatment
Essential fructosuria	Fructokinase	229800	Asymptomatic	*KHK*/AR	No treatment
Hereditary fructose intolerance	Aldolase B	229600	Abdominal pain, nausea, hypoglycemia symptoms, shock-like syndrome after fructose ingestion	*ALDOB*/AR	Withdrawal of all sources of fructose (food, drugs, liquids, parenteral infusions) Intravenous glucose for hypoglycemia Supplementation with folate and vitamin C
FBPase deficiency	FBPase	229700	Life-threatening episodes of hypoglycemia, coma triggered by a febrile episode, fasting or large amount of fructose ingestion (~1 g/kg BW)	*FBP1*/AR	Avoiding catabolic triggers Frequent feeding (use of slowly absorbed cornstarch) but to avoid overfeeding Hypoglycemia correction with oral +/− IV glucose In the absence of triggers no CH supplements needed Restriction of fructose, sucrose and sorbitol

AR: autosomal recessive, BW: body weight, CH: carbohydrate, FBPase: fructose-1,6-bisphosphatase.
